# Agency of students participating in extracurricular activities and their interaction with parents in the context of the pandemic

**DOI:** 10.3389/fpsyg.2025.1484789

**Published:** 2025-05-21

**Authors:** Mikhail Goshin, Dmitry Grigoryev, Pavel Sorokin, Polina Bochkareva

**Affiliations:** ^1^Pinsky Center of General and Extracurricular Education, Institute of Education, National Research University Higher School of Economics, Moscow, Russia; ^2^Center for Socio Cultural Research, National Research University Higher School of Economics, Moscow, Russia; ^3^Laboratory for Human Capital and Education Research, Institute of Education, National Research University Higher School of Economics, Moscow, Russia

**Keywords:** agency, extracurricular activities, parental strategies, COVID-19 pandemic, education

## Abstract

This article investigates the relationships between strategies of parental involvement in education and manifestations of children’s agency during the pandemic seen as a potentially harmful and stressful context, requiring agency for sustaining well-being to a greater extent than before COVID-19. Data for the study were obtained through an online survey of students engaged in extracurricular activities, about the transition to distance learning and self-isolation during the pandemic. To elucidate the understanding of differences among respondents regarding changes in their interaction with parents, latent profile analysis was applied. It was found that joint activity between children and parents can be associated with the formation of a special type of agency, which is called ‘cooperative agency’, while parents providing children with freedom and facilitating support are associated with other behavioral characteristics of the child, i.e., ‘autonomous agency’. At the same time, the absence of interaction with parents, as well as parents’ display of strict control, do not contribute to successful adaptation to crisis conditions.

## 1 Introduction

In modern times, the education system is faced with new challenges due to emerging socio-economic trends. A pressing issue is the development of ‘transformative agency’, which involves the ability to proactively influence one’s social environment and create new communities and forms of interaction in various spheres of public life (Udehn, 2002; Kirby, 2020; Nunes et al., 2023). Proactive behavior refers to the efforts made by individuals to develop and enhance their overall resources, which helps them progress towards challenging objectives and personal development. They view difficult circumstances as opportunities for growth and improvement. By cultivating of agency, individuals can perceive stress or challenging situations as chances to enhance themselves and take ownership of their decisions and behaviors (Schwarzer, 2001). Agency becomes especially important and discussed under conditions of crisis and stress, of which the pandemic COVID-19 is a good example.

The extracurricular activities (ECA) have a high potential in terms of the formation and development of relevant personal qualities and behavioral patterns. Participation of schoolchildren in ECA contributes to the formation of perseverance, independence, cognitive motivation, self-confidence, creativity, and social activity of children and youths (Farkas, 2003; Fletcher et al., 2003; Baker, 2008). There are two main reasons underlying the importance of ECA for agency issues. Firstly, it is a personal choice of programs with a relatively high degree of freedom, in comparison with the basic school curriculum (Lareau and Weininger, 2008). Secondly, it is the content features of ECA, which are characterized by an emphasis on the creation of educational products, including projects, which allows the student to develop and show the ‘agent potential’ to a greater extent.

Against the well-researched effects of ECA for various aspects of individual development, the study of the contribution of ECA to the formation of the ability to engage in proactive action is limited (Carbonaro and Maloney, 2019). The factors and conditions for the formation of this ability, including the influence of the family and the peculiarities of parental participation in the education of schoolchildren, have not been sufficiently explored (UNESCO, 2020).

The present paper analyses the relationship between strategies of interaction with parents and manifestations of agency among children engaged in ECA under the restrictions caused by the COVID-19 pandemic. This focus of the study is justified by the fact that the combination of these factors created a unique opportunity for an in-depth exploration of the complex and multifaceted construct of agency.

## 2 Literature review

### 2.1 Agency of schoolchildren and participation in extracurricular activities

There are numerous approaches to defining agency. Agency implies the independence of the individual, their ability to act according to their own desires and beliefs. However, agency is always in interaction with the context in which an individual acts. The structural opportunities available to a person play an important role in the realization of agency. These opportunities can either limit or, on the contrary, expand the space for action, influencing the degree of freedom that a person feels in their actions (Bazzani, 2023). Therefore, agency can be characterized as a dynamic process that includes both the internal resources of the individual and the external conditions that determine their behavior (Crossley, 2021; Torrance and Froese, 2011). We consider agency as a person’s ability to act as an independent agent, making a free and conscious choice; to influence the surrounding social environment, transforming existing and creating new forms of interaction in various spheres of social life (Sorokin, 2023).

In the context of school education and socialization of a young person, the development of agency plays a particularly important role, since it is during this period that basic life attitudes, motivation to study and abilities to interact with the world around us are formed (Gallagher et al., 2019). However, unlike school education, the sphere of ECA contains much more opportunities for the manifestation of ‘free’, agentic action. The implementation of ECA aims to enhance the students’ potential and develop character values (Narimo and Irawan, 2018). These activities serve as valuable learning experiences that contribute to the development of students’ personalities. Students can enhance their cognitive, affective, and psychomotor skills (Abidin, 2019).

Engaging schoolchildren in ECA enables them to enhance organizational skills, acquire valuable knowledge, tackle challenges, and foster important values (Patle, 2024; Javed and Srivastava, 2024). Participating in ECA has a positive impact on overall well-being as it allows individuals to pursue their personal interests, actively engage with a sense of purpose, and develop essential skills and competencies. Different factors, such as the type of involvement (social, physical, or cognitive), the level of engagement (frequency, intensity, consistency, and continuity), and the variety of activities participated in, characterize participation in ECA (Oberle et al., 2019). Arts and leadership programs frequently focus on empowering teenagers to strive for objectives through involvement in significant individual or group endeavors, such as creating artwork, organizing a performance, coordinating an event, or making a positive impact on their community. Several studies have shown that the success of these initiatives ultimately hinges on the students’ agency (Handy et al., 2020; Syaharuddin et al., 2020; Mawaddah et al., 2022). However, in general, the relationship between the participation of schoolchildren in ECA and the formation of agency remains under studied.

### 2.2 Parental involvement in education and children’s agency

Research into the issues of children’s agency in conjunction with strategies reflecting the parental participation in education is of particular relevance. Parental participation has been actively studied in various countries (Epstein, 2007; Goshin et al., 2021). It involves both participation in school life, and the creation of home environment that maximizes effective learning. This environment includes stimulating intellectual activity, discussing and helping to solve difficulties, assisting with homework, promoting the child’s aspirations related to self-realization, and civic position.

The relationship between the parental participation in education and the involvement of schoolchildren in ECA is very heterogeneous. Thus, teenagers whose parents express approval and provide moral and material support usually participate in more ECA, achieve better results, and enjoy these activities more. However, parental involvement in the form of pressure or coercion can significantly limit the desire and ability of adolescents to participate in ECA (Anderson et al., 2003; Goshin et al., 2021). Excessive parental pressure can undermine the interest and initiative of students, leading to a negative attitude towards these types of activities (Ashbourne and Andres, 2015). Children whose parents intervened more often to give direction, make corrections or suggestions - even though they properly completed the task - had more difficulties regulating their behavior and emotions, performed worse on tasks that measured ‘delayed reward’, and skills related to switching between competing demands for their attention. Too much direct parental involvement can negatively affect children’s abilities to control their own attention, behavior, and emotions (Obradović et al., 2021). When parents allow children to play a leading role in their interaction, children develop self-regulation skills and gain independence.

Children can show agency differently in different spheres, i.e., with parents, teachers, and peers (Gurdal and Sorbring, 2018). When dealing with adults such as parents or teachers, agency may be demonstrated by ignoring or refusing (Trust and Whalen, 2020). But with peers, children tend to employ democratic solutions to express their agency. The study (Kumpulainen et al., 2019) demonstrates that the child’s agency is co-created through joint activities between the child and adult. This research emphasizes the importance of children being accountable for joint rules, highlighting how their agency is intertwined with these rules. The trust between parents and children is crucial for fostering the opportunities for children to express their agency.

Thus, the development of schoolchildren agency also depends largely on parental strategies, but the factors and conditions for developing this ability, especially regarding the influence of the family, are insufficiently studied. Literature on issues of agency pays serious attention to childhood and the role of parents (Abebe, 2019). However, researchers focus on violence and resistance to it (Cavazzoni et al., 2020), children’s rights (Oswell, 2013), and their physical well-being and health (Victor et al., 2013), rather than on children’s agency in education and the related factors of the family environment.

### 2.3 Lessons learned from the COVID-19 pandemic

In 2020, the introduction of emergency measures to prevent the spread of COVID-19, the suspension of face-to-face education, and the transfer of most classes to a distance format became a real challenge for the global education system. Families found themselves in conditions of increased stress, associated with concern for health, the risk of losing their jobs, and the need to organize and continue their children’s education at home (Kalil et al., 2020; Weaver and Swank, 2021). This situation particularly affected schoolchildren’s participation in ECA, which is much more oriented towards live communication between children and educators and like-minded people, practical activities, compared to school classes (Gushchina, 2021). During isolation, children’s participation in ECA decreased (Ilari et al., 2021), which also led to a decrease in academic motivation among students.

As a result of the pandemic, the role and responsibility of students and their families in the educational process have increased (Kalil et al., 2020; Weaver and Swank, 2021). New and unique conditions have emerged for the manifestation of agency and, therefore, for their study and analysis. In these conditions, the study of the agency of children engaged in ECA, in conjunction with strategies of parental participation in education, are of relevance.

The experience of the pandemic has highlighted the important role of children’s agency, particularly their ability to act independently. It is not just the ability to do what was previously provided by additional tools of control, but also the ability to do it differently; for example, the ability to independently learn and practically apply new educational content without losing the motivation to learn (Dwivedi et al., 2020). Adaptation to new conditions in times of significant societal changes (like during the pandemic) always requires, to some extent, the development of special rules, including within the family circle (Emirbayer and Mische, 1998). These rules could relate to the time distribution between leisure, housework and other activities, as well as to a wide range of strategies and tactics regarding both school and extracurricular education in a distance format. For example, it may deal with daily routines, mutual responsibilities regarding everyday issues, and much more (Amadasi and Baraldi, 2022; Rogers, 2022; Ignjatović, 2022; Fauziddin et al., 2021). We can view this process as an expression of agency, which assumes the ability of families to find effective solutions in response to «grand challenges», based on their unique needs and circumstances. In conditions of ‘de-structuring’ and the sharply exacerbated problem of the breakdown of familiar solidarity and cohesion systems of the 20th century (Amadasi and Baraldi, 2022; Sorokin and Popova, 2021, 2022), including in education, one of the key indicators of agency can be considered the ability to proactively create new communities and groups (Ling and Dale, 2014; Newman and Dale, 2005). This has become particularly relevant during the pandemic, albeit in the online space.

The analysis of the interaction between parents and children in the context of ECA has broad potential for understanding how children’s agency develops under conditions of stress and uncertainty. Studying this phenomenon during a pandemic opens new horizons for scientific research, providing an opportunity for a systematic approach to understanding the mechanisms of forming a predisposition to proactive behavior in the educational environment, including understanding the role of a family. The purpose of this study is to demonstrate how the models of interaction between schoolchildren and their parents are interrelated with the manifestations of children’s agency during the covid-19 pandemic.

Accordingly, we address the following research questions:

1. How has the pandemic altered the dynamics between schoolchildren and their parents?
2. What percentage of families developed new rules to adapt to the pandemic, and how did this compare to families who employed alternative strategies for parent–child interaction? If new rules were established, what was their origin?
3. Among families with different interaction strategies, what proportion of students created online groups on topics related to education or other subjects during the quarantine?
4. What challenges did students from families with varying parental strategies encounter, and what opportunities did they see for themselves in the new situation? Which strategies proved to be the most effective for successful adaptation to changed conditions?

## 3 Materials and methods

Data were obtained from the surveys of schoolchildren (*N* = 16,666) on the transition to a remote form of education during self-isolation (May 2020). The sample includes representatives from all federal districts. The level of Internet penetration in Russia is high and relatively homogeneous with more than 80% of the population covered. Taking into account the large sample size, the survey results can be considered representative for Russia.

Children aged 7 to 18 participated in the survey, with the proportion of children aged 7 to 10 years old being 36.7%, the proportion of younger teenagers aged 11 to 14 years old being 43.1%, and the proportion of older teenagers aged 15 to 18 years old being 20.2%. Girls accounting for 63% of those surveyed, which is typical for extracurricular education in general. Informed consent from the school administration was obtained when distributing the questionnaires, including information that the link to the questionnaire is sent exclusively to children who have reading skills and the ability to complete online surveys. In Russia, most children already have reading skills when they enter the first grade.

The questionnaire consisted of 22 inquiries and was specially designed to study the participation of schoolchildren in ECA during the transition to remote learning in the pandemic and self-isolation regime. The questions regarding agency were compiled based on the literature data on the problem of children’s agency, as well as current discussions in the social sciences (Emirbayer and Mische, 1998; Amadasi and Baraldi, 2022; Rogers, 2022; Fauziddin et al., 2021; Amadasi and Baraldi, 2022; Sorokin and Popova, 2021, 2022; Ling and Dale, 2014). The survey delved into topics like the establishment of quarantine adaptation rules within families *(Have clear rules been established in your family according to which you continue your education during quarantine?)* and their origins *(Where did the new rules for continuing your education and organizing life during quarantine come from?)*, as well as the child’s social transformative agency measured through their creation of online communities related to education or other subjects *(Did you create on your own (or co-initiate the creation of) online communities during the quarantine period, focusing on education or other topics?)*. The focus on digital communities and groups was due to the heightened importance of remote communication methods. The survey also evaluated the impact of ECA during the pandemic and the success of adaptation to new conditions among the respondents.

To assess the changes in respondents’ interaction with parents and strategies of parental participation in children’s education during the quarantine, a corresponding question was included in the questionnaire evaluating various options for joint actions and their dynamics *(How has your communication with parents changed due to self-isolation during the coronavirus pandemic?)*Our study utilized the data-driven approach, also known as the ‘bottom-up methodological approach’, to analyze the data. We employed latent profile analysis (LPA; Grigoryev and van de Vijver, 2017), which is an exploratory technique that utilizes the maximum likelihood method to establish an internal latent structure in the sample. LPA allowed us to determine the observed nature of the responses and classify the study participants based on certain initially implicit characteristics. LPA enabled us to group the respondents based on their answers to the question about changes in the nature of interaction with parents due to self-isolation during the pandemic. LPA was conducted using the Mplus programs in the statistical environment R. To identify the best model, a special hierarchical selection algorithm (Akogul and Erisoglu, 2017) was automatically used, based on the following fit indices: Akaike Information Criterion (AIC), Approximate Weight of Evidence Criterion (AWE), Bayesian Information Criterion (BIC), Classification Likelihood Criterion (CLC), and Kulback-Leibler Information Criterion (KIC). This selection algorithm showed that the best model was a five-latent profile model, in which the variances and covariances of variables were considered equivalent across all profiles. The entropy index was 0.988. Additionally, based on the results of this grouping, each profile included an adequate minimum number of respondents, allowing us to proceed with the obtained classification and further analyze the differences between profiles.

Next, questions about children’s agency (the establishment of quarantine adaptation rules within families and their sources, the child’s creation of online communities related to education or other subjects, and the success of adaptation to new conditions) were juxtaposed with the defined five categories of respondents by making crosstabs (Tysinger et al., 2016). The statistical significance level was evaluated by the chi-square test in software package IBM SPSS Statistics 27.0.

## 4 Results

**Figure 1:**
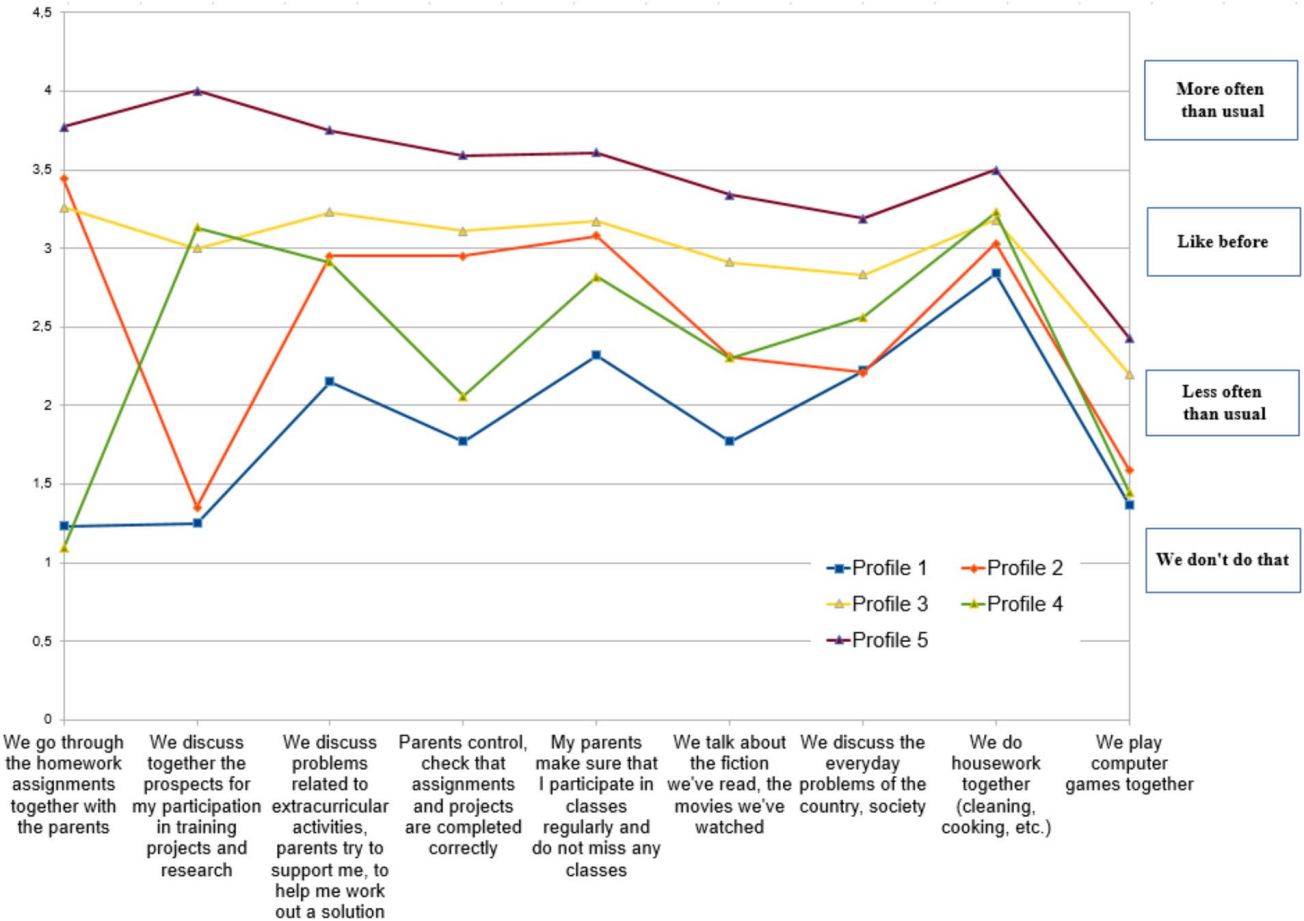
Characteristics of five latent profiles of respondents.

Children’s interaction with parents underwent various changes during the pandemic and the transfer of education to a distance format. LPA divided the respondents into five categories (Figure 1). The category *‘Weakly Involved Parenting’ (Profile 1, 12.%)*, is characterized by the lowest level of interaction with parents. For most positions, they usually indicated “We do not do this,” except for joint household chores. The pandemic did not significantly change this situation. Children belonging to the group *‘Controlling Parenting’ (Profile 2, 10.4%)* began discussing assignments slightly more frequently with their parents, besides which their parents tend towards controlling strategies. However, they almost never discuss educational projects or research opportunities with their parents, meaning strategic perspective-oriented activity is not supported. The largest category is *‘Total Engagement’ (Profile 3, 48.2%);* their parents are engaged in various forms of interaction with children, maintaining the same frequency as before. A small category *‘Support for Individual Project Activities’ (Profile 4, 4.3%)* is characterized by joint discussions with parents about prospects for participation in educational projects and research. Parental control and collaborative work on tasks with parents are not typical for this group. Representatives of the category *‘Complex Increased Involvement’ (Profile 5, 25.1%)* have become much more frequent in interacting with parents across nearly all positions since the onset of the pandemic, except for computer games played together. Most notably, there has been an increase in the frequency of jointly discussing future participation in educational projects and research endeavors.

The respondents belonging to different age levels are represented differently in these latent profiles. The proportion of the youngest study participants (5–10 years old) is maximal in Profiles 5 and 2 (45.6 and 41.7%, respectively) and minimal in Profile 4 (8.0%). Accordingly, Profile 4 is characterized by the largest proportion of older respondents aged 15–18 years (46.9%). The least number of high school students is in Profiles 2 and 5 (12.6 and 14.0%, respectively).

Younger adolescents (11–14 years old), who occupy an intermediate position, were distributed more evenly among the profiles. Their proportion is highest in Profile 1 (47.4%) and lowest in Profile 5 (40.5%).

**Figure 2:**
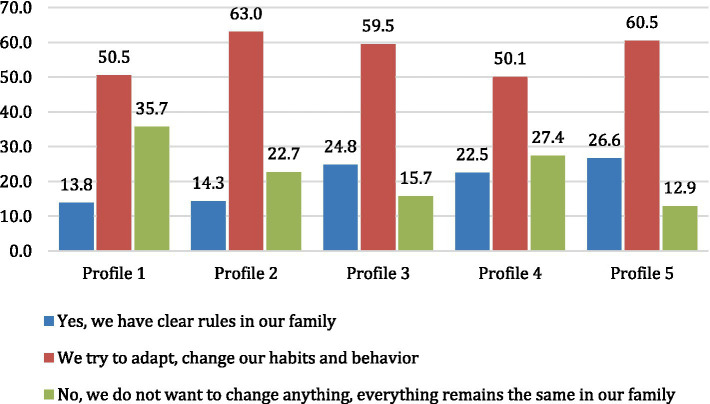
The presence of rules in the family that help to adapt to new conditions, % (χ^2^(df) = 562.219 (8); *p* < 0.001).

We examined how families from different respondent categories adapted to new conditions during the pandemic. Families in profiles 3 and 5 were most successful in developing rules to continue their children’s education during quarantine. This involved increasing interactive activities with parents that were previously less frequent (Figure 2). Families in profiles 1 and 4, where parents are less involved in their children’s education or give them more freedom, were less likely to report the development of such rules.

**Figure 3:**
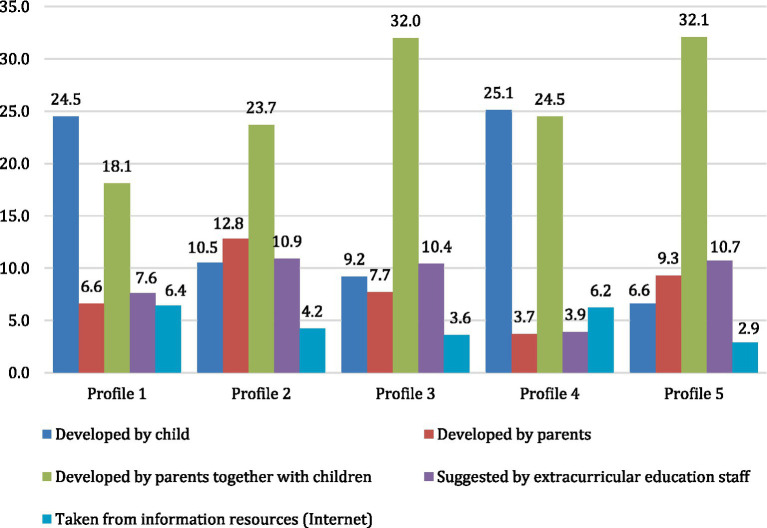
Sources of rules that help to adapt to new conditions, % (χ^2^(df) = 717.099 (24); *p* < 0.001).

Regarding the origin of the new rules (Figure 3), it was found that the students in profiles 1 and 4 were more likely to create the rules themselves. In contrast, profiles 3 and 5 had fewer families where the children developed the rules independently, with most rules being created by parents or jointly with children, respectively. It appears that profiles 1 and 4 demonstrate autonomous agency, while profiles 3 and 5 exhibit cooperative agency through collaborative efforts between parents and children.

The data suggests that there is a noticeable increase in the percentage of students in profiles 1 and 4 who create rules independently as they progress from primary to high school (Table 1). Profiles 3 and 5 have a consistent proportion of students who develop rules jointly with their parents, which accounts for about one third of the overall sample and does not vary significantly with age. It is worth noting that these trends remain stable across different age groups, indicating the reliability of the profile categorization.

**Table 1:** Sources of rules that help to adapt to new conditions in different age groups, %.

The sources of rules	Profile 1	Profile 2	Profile 3	Profile 4	Profile 5
5–10 years old (χ^2^(df) = 96.280 (24); *p* < 0.001)					
Developed by child	8.6	4.0	2.9	8.5	2.9
Developed by parents	14.9	19.7	11.1	14.9	11.1
Developed by parents together with children	21.6	24.0	34.0	34.0	33.1
Suggested by extracurricular education staff	15.3	13.4	11.7	6.4	11.8
Taken from information resources (Internet)	3.6	2.5	2.7	6.4	2.1
11–14 years old (χ^2^(df) = 229.029 (24); *p* < 0.001)					
Developed by child	21.1	12.1	9.2	22.0	7.7
Developed by parents	7.8	8.2	6.3	4.2	8.4
Developed by parents together with children	19.0	25.0	32.0	24.6	31.9
Suggested by extracurricular education staff	7.5	9.2	10.0	3.0	10.2
Taken from information resources (Internet)	6.0	5.2	3.0	6.1	2,0.2
15–18 years old (χ^2^(df) = 134.387 (24); *p* < 0.001)					
Developed by child	34.2	24.9	21.6	30.9	14.9
Developed by parents	2.3	7.2	4.3	1.5	6.0
Developed by parents together with children	15.7	18.2	28.0	22.5	30.7
Suggested by extracurricular education staff	4.9	9.4	8.4	4.4	8.5
Taken from information resources (Internet)	7.9	5.5	6.3	6.2	7.1

**Figure 4:**
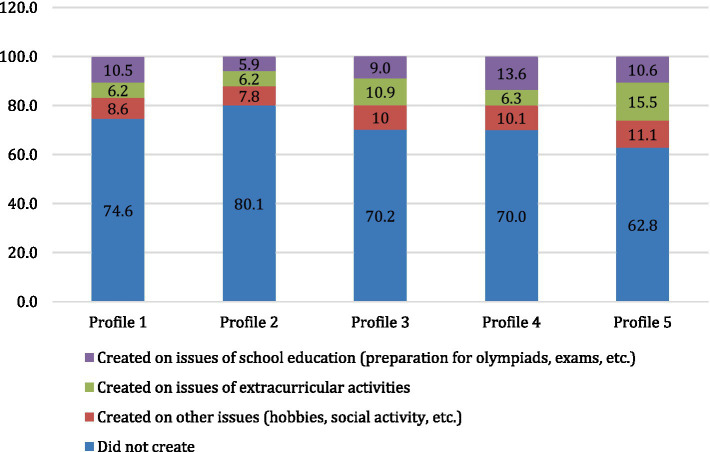
The proportion of students who created (or co-initiated the creation of) communities on the Internet in quarantine conditions, % (χ^2^(df) = 247.278 (12); *p* < 0.001).

Turning our attention to the expression of social-agent activity across different profiles (Figure 4), we can observe that profile 5 participants frequently engage in group and community formation, both within educational contexts and beyond. This aligns with the cooperative agency trait of this group in terms of jointly developing new family rules with parents. Conversely, profile 2 individuals exhibit the least amount of social-agent behavior, likely due to their parents’ strict controlling approach.

The proportion of children engaged in community’s formation shows some variations depending on the age. Thus, profile 5 participants show a significant increase in social-agent activity as they grow older. While only 27.2% of this profile’s members are involved in creating communities between the ages of 5 and 10, this number increases to over half (51.9%) between the ages of 15 and 18. In contrast, other profiles do not show such a substantial increase in the proportion of children engaged in social-agent behavior as they age.

**Figure 5:**
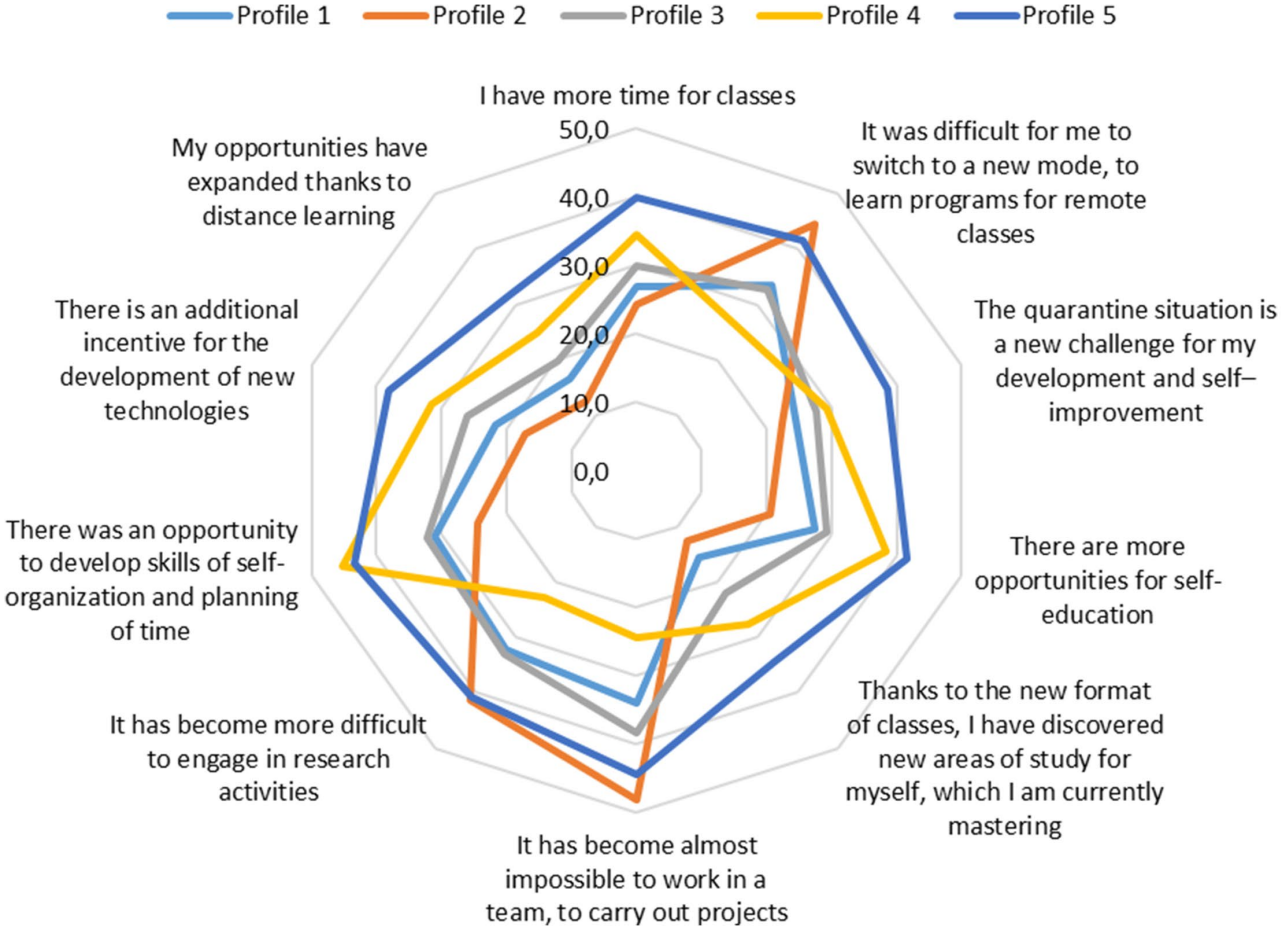
The proportion of respondents who expressed perfect agreement with the statements about the new conditions of ECA in quarantine, %.

In terms of adaptation to new conditions and their effects (Figure 5), there are significant differences between latent profiles. Representatives of profile 5 are much more likely to note positive aspects and expanded capabilities in new conditions. They frequently express complete agreement with statements such as ‘I have more time for classes’, ‘The quarantine situation is a new challenge for my development and self-improvement’, ‘There are more opportunities for self-education’, and similar. However, these individuals also frequently noted the difficulties of adapting to new conditions, such as ‘It was difficult for me to switch to a new regime, master programs for remote classes’, ‘It became almost impossible to work in a team, to carry out projects’, and ‘It became more difficult to engage in research activities.’ Representatives of profile 2 are the least likely to see the expansion of their capabilities in the new conditions, which is consistent with their reports of facing difficulties. Representatives of profile 4 also see positive aspects for their development in the new situation. Notably, these individuals are less likely to agree with statements about obstacles despite their high degree of agreement with positive statements.

## 5 Discussion

The presented study aims to capture the relationships between the manifestation of agency by students, the effectiveness of adapting to new learning conditions that have arisen because of the pandemic, and strategies for interacting with parents, which lie in two dimensions: on the one hand, the presence of various forms of joint activity with parents, and on the other hand, changes that have occurred because of the pandemic. This perspective presented the opportunity for a fresh look at parental involvement strategies in education in relation to children’s success, in terms of the manifestation of agentic, proactive behavior aimed at adapting to new conditions, including the ability to see opportunities for self-development in them and take responsibility for things happening.

First and foremost, it is important to note that we observe the development of rules more often in families where parents are highly involved in interacting with their children, particularly in cases where different forms of joint activities occur more frequently, to help structure life, adapt to new conditions, and continue education during the quarantine (Profile 5). Rules are less present in families where parental involvement in education is minimal. However, for profiles with minimal parental involvement (Profile 1 and Profile 4), children tend to develop rules independently, demonstrating proactive adaptation for current situation of disorganization, and forming rules on their own. This activity is similar in its characteristics to the model of proactive coping behavior described in the literature (Zimmerman and Cleary, 2006), which integrates planning and preventive strategies with proactive self-regulation to achieve goals.

The described situation vividly demonstrates the manifestation of different types of agent behavior among children: representatives of Profile 1 and Profile 4 exhibit autonomous agency, while Profiles 3 and 5 exhibit cooperative agency. It is evident that high parental involvement in their children’s education and joint activities contribute to the expression of cooperative agency, while providing significant freedom (Profile 1) or freedom with facilitating support (Profile 4) leads to autonomous agency, which is consistent with the results of previous studies (Guest and Schneider, 2003; Goshin et al., 2022). Interestingly, respondents from Profile 1 and Profile 4 more often noted that they obtained rules from information resources (Internet). The ability to independently search for any necessary information is fully consistent with the strategy of autonomous agency.

The representatives of Profile 2, who are most susceptible to parental control, often reported that the rules were developed by their parents. This may be one manifestation of authoritarian parenting strategy, which has been shown to have a negative relationship with children’s proactive behavior, confirming the results of previous studies (Ashbourne and Andres, 2015). This is consistent with the idea of cooperative agency and the participation of respondents in social-agent activities, in this case manifested in the creation of communities on the Internet. Learning communities that demonstrated a cooperative agent strategy together with their parents were more likely to create such groups than those who experienced maximum parental control. Children who experienced maximum parental control were less likely to engage in such activities.

The distribution of profiles by age is quite explainable, considering that adolescence is accompanied by rapid development in all areas, including cognitive and emotional domains. Consequently, as young people grow older, they develop greater ability to make independent decisions, and their autonomy from parents increases (Nunes et al., 2023). Older teenagers no longer require total parental engagement in their education, although such engagement is highly valued in elementary school. This trend also explains the increasing proportion of students who independently developed rules as they grew older. However, the question of how agency manifests itself at different ages in relation to parental strategies requires further detailed studies.

Representatives of Profile 4 and Profile 5 often talk about expanding opportunities and new perspectives in the current situation. This indicates that both autonomous and cooperative agency strategies are effective in terms of adapting to new conditions and seeing difficult situations as a stimulus for self-development (Zimmerman and Cleary, 2006). Moreover, the cooperative strategy is slightly more successful in this regard. However, representatives of Profile 5, who are characterized by cooperative agency, more often mention barriers, difficulties in adaptation, and challenges in continuing research and teamwork at all age levels, while for Profile 4, characterized by autonomous agency, these obstacles were not so significant. Perhaps these children are more oriented towards individual forms of work, for which the transition to distance learning was not accompanied by significant difficulties.

It should be noted that representatives of Profile 4 demonstrate a higher degree of adaptation compared to Profile 1 in almost all positions. Representatives of Profile 2 more often note the difficulties they have faced and less often report expanding their opportunities. This profile is characterized by a high degree of parental involvement, manifested in control and joint task execution. However, as in Profile 1, there is almost no joint discussion with parents about the prospects of participating in educational projects and research. The results obtained confirm data from other sources about the destructive influence of strict parental control and excessive intervention in the child’s educational activities (Anderson et al., 2003; Goshin et al., 2021). The most differentiating feature in the interaction strategies between children and parents is the characteristic of joint discussion with parents about the prospects of participating in educational projects and research. This is a special form of activity that has independent value and provides an important stimulus for self-development. These results also confirm that trust between parents and children is crucial for creating opportunities for children to express their independence (Kumpulainen et al., 2019).

## 6 Limitations and future research

This study was conducted in the context of pandemic, which imposes certain limitations. Firstly, conducting the survey exclusively online may lead to a bias towards the group of children who have access to the necessary devices and a stable internet connection. Although most children today fall into this category, there is still a risk that the study’s results may not fully reflect the opinions of all schoolchildren. Secondly, conducting the research during lockdown, although it reveals several important aspects of schoolchildren’s agency, is perceived as a snapshot reflecting the specifics of this unique period when everyday life was drastically altered. This circumstance may limit the generalizability of the data obtained to a broader timeframe. We focus exclusively on relations between parents and children in terms of educational activities and related changes connected with adaptation to pandemic; we do not take into account any other possible transformations on family life, though they are also likely to happen in such circumstances but are out of scope of the present research.

Accordingly, it is advisable to conduct further research on the conditions and effects of children’s agency manifestations, with a focus on the field of ECA, preferably in a longitudinal mode, outside of lockdown. It makes sense to include additional variables that could help explain the results but were not included in this study. For example, children and families’ socioeconomic status may influence their participation in ECA and the manifestation of agency. Deepening research in this area will contribute to a more comprehensive understanding of children’s development and adaptation in a changing world.

## 7 Conclusion

There is a significant correlation between children’s interaction strategies with their parents regarding educational issues and their agency during the pandemic. Joint activities between children and parents are linked to cooperative agency, while providing children with freedom and support is associated with autonomous agency. Families where parents were highly involved in interacting with their children, especially through joint activities, were more likely to develop rules that helped structure life and adapt to new conditions during the quarantine. These children were also more likely to create communities and groups online, demonstrating a cooperative agency strategy with their parents. Conversely, children who experienced strict parental control were less likely to engage in proactive behavior. Complete lack of interaction or strict parental control did not contribute to successful adaptation in crisis conditions. Finally, discussing educational projects and research prospects with parents was deemed crucial for expanding opportunities and promoting proactive behavior in stressful conditions.

## Data Availability

The raw data supporting the conclusions of this article will be made available by the authors, without undue reservation.

## References

[ref1] Abebe T. (2019). Reconceptualising children’s agency as continuum and interdependence. Soc. Sci. 8:81. doi: 10.3390/socsci8030081

[ref2] Abidin M. (2019). Penerapan Pendidikan Karakter Pada Kegiatan Ekstrakurikuler Melalui Metode Pembiasaan. J. Kependidikan 12, 183–196. doi: 10.30863/didaktika.v12i2.185

[ref3] Akogul S. Erisoglu M. (2017). An approach for determining the number of clusters in a model-based cluster analysis. Entropy 19:452. doi: 10.3390/e19090452

[ref4] Amadasi S. Baraldi C. (2022). The child as a medium. Breakdown and possible resurgence of children’s agency in the era of pandemic. Childhood 29, 561–577. doi: 10.1177/09075682221098156, PMID: 36408481 PMC9647231

[ref5] Anderson J. C. Funk J. B. Elliott R. Smith P. H. (2003). Parental support and pressure and children’s extracurricular activities: relationships with amount of involvement and affective experience of participation. J. Appl. Dev. Psychol. 24, 241–257. doi: 10.1016/S0193-3973(03)00046-7

[ref6] Ashbourne D. Andres L. (2015). Athletics, music, languages, and leadership: how parents influence the extracurricular activities of their children. Can. J. Educ. 38, 1–34. doi: 10.2307/canajeducrevucan.38.2.09

[ref7] Baker C. N. (2008). Under-represented college students and extracurricular involvement: the effects of various student organizations on academic performance. Soc. Psychol. Educ. 11, 273–298. doi: 10.1007/s11218-007-9050-y

[ref8] Bazzani G. (2023). Agency as conversion process. Theory Soc. 52, 487–507. doi: 10.1007/s11186-022-09487-z, PMID: 37287699 PMC10241693

[ref9] Carbonaro W. Maloney E. (2019). Extracurricular activities and student outcomes in elementary and middle school: causal effects or self-selection? Socius 5, –237802311984549. doi: 10.1177/2378023119845496, PMID: 40270978

[ref10] Cavazzoni F. Fiorini A. Veronese G. (2020). Well-being and life satisfaction in children living in contexts of political violence: a narrative literature review. Child Youth Care Forum 52, 1–24. doi: 10.1007/s10566-022-09678-w

[ref11] Crossley N. (2021). A dependent structure of interdependence: structure and agency in relational perspective. Sociology 56, 166–182. doi: 10.1177/00380385211020231, PMID: 40270978

[ref12] Dwivedi Y. K. Hughes D. L. Coombs C. Constantiou I. Duan Y. Edwards J. S.. (2020). Impact of COVID-19 pandemic on information management research and practice: transforming education, work and life. Int. J. Inf. Manag. 55:102211. doi: 10.1016/j.ijinfomgt.2020.102211

[ref13] Emirbayer M. Mische A. (1998). What is agency? Am. J. Sociol. 103, 962–1023. doi: 10.1086/231294

[ref14] Epstein J. (2007). Connections count: improving family and community involvement in secondary schools. Princ. Leadersh. 8, 16–22.

[ref15] Farkas R. (2003). Effects of traditional versus learning-styles instructional methods on middle school students. J. Educ. Res. 97, 42–51. doi: 10.1080/00220670309596627

[ref16] Fauziddin M. Mayasari D. Rizki M. (2021). Effective learning for early childhood during global pandemic. Al-Ishlah J. Pendidikan 13, 515–522. doi: 10.35445/alishlah.v13i1.458

[ref17] Fletcher A. C. Nickerson P. Wright K. L. (2003). Structured leisure activities in middle childhood: links to well-being. J. Community Psychol. 31, 641–659. doi: 10.1002/jcop.10075

[ref18] Gallagher M. W. Long L. J. Richardson A. D’Souza J. M. (2019). Resilience and coping in cancer survivors: The unique effects of optimism and mastery. Cognit. Ther. Res. 43, 32–44. doi: 10.1007/s10608-018-9975-9, PMID: 31223177 PMC6586435

[ref19] Goshin М. Dubrov D. Kosaretsky S. Grigoryev D. (2021). The strategies of parental involvement in adolescents’ education and extracurricular activities. J. Youth Adolesc. 50, 906–920. doi: 10.1007/s10964-021-01399-y, PMID: 33528703

[ref20] Goshin M. E. Sorokin P. S. Kosaretsky S. G. (2022). Agency of Schoolchildren in the changing educational context during COVID-19 pandemic: sources, manifestations, and effects. Monitor. Public Opin. Econ. Soc. Changes J. 5, 394–417. doi: 10.14515/monitoring.2022.5.2145

[ref21] Grigoryev D. van de Vijver F. (2017). Acculturation profiles of Russian-speaking immigrants in Belgium and their socio-economic adaptation. J. Multiling. Multicult. Dev. 38, 797–814. doi: 10.1080/01434632.2016.1268145

[ref22] Guest A. Schneider B. (2003). Adolescents’ extracurricular participation in context: the mediating effects of schools, communities, and identity. Sociol. Educ. 76, 89–109. doi: 10.2307/3090271

[ref23] Gurdal S. Sorbring E. (2018). Children’s agency in parent–child, teacher–pupil and peer relationship contexts. Int. J. Qual. Stud. Health Well Being 13:1565239. doi: 10.1080/17482631.2019.1565239, PMID: 30709328 PMC6366412

[ref24] Gushchina T. N. (2021). Features of social and pedagogical support of children with the use of distance learning tools in the organization of additional education. Innov. Obrazovanii 8, 100–110.

[ref25] Handy M. R. N. Mutiani M. Putra M. A. H. Jumriani J. (2020). The religious values in tradition of Batahlil in Banjar Pahuluan community. Kalimantan Soc. Stud. J. 2, 39–47. doi: 10.20527/kss.v2i1.2462

[ref26] Ignjatović G. (2022). Systemic agency in education in extraordinary circumstances: challenges encountered by LF Niš during the COVID-19 pandemic and opportunities for developing systemic agency in legal education. Zb. Rad. Prav. Fak. Nis. 61, 65–97. doi: 10.5937/zrpfn1-42724

[ref27] Ilari B. Cho E. Li J. Bautista A. (2021). Perceptions of parenting, parent-child activities and Children’s extracurricular activities in times of COVID-19. J. Child Fam. Stud. 31, 409–420. doi: 10.1007/s10826-021-02171-3, PMID: 34840489 PMC8611177

[ref28] Javed I. Srivastava A. K. (2024). An analysis of role of extracurricular activities (ECA) in higher education. Rev. Rev. Index J. Multidiscip. 4, 66–73. doi: 10.31305/rrijm2024.v04.n01.008

[ref29] Kalil A. Mayer S. Shah R. (2020). Impact of the COVID-19 crisis on family dynamics in economically vulnerable households; Becker Friedman Institute for Economics Working Paper. Chicago, IL, USA: University of Chicago, 1–30.

[ref30] Kirby P. (2020). Children’s Agency in the Modern Primary Classroom. Child. Soc. 34, 17–30. doi: 10.1111/chso.12357

[ref31] Kumpulainen K. Sairanen H. Nordström A. (2019). “Young Children’s Agency in Their Digital Media use in the sociocultural contexts of their homes: a case study from Finland” in Crianças, famílias e tecnologias. Que desafios? Que caminhos? eds. Brito R. Dias P. (Lisboa: Centro Interdisciplinar de Estudos Educacionais), 150–170.

[ref32] Lareau A. Weininger E. B. (2008). “Class and the transition to adulthood” in Social class: How does it work? eds. Lareau A. Conley D. (New York: Russell Sage Foundation), 118–151.

[ref33] Ling C. Dale A. (2014). Agency and social capital: characteristics and dynamics. Community Dev. J. 49, 4–20. doi: 10.1093/cdj/bss069

[ref34] Mawaddah A. Syaharuddin M. Abbas E. W. Jumriani. (2022). Extracurricular activities PMR (red cross teen) at Banua South Kalimantan bilingual boarding high school makes students with character. Kalimantan Soc. Stud. J. 3, 91–100. doi: 10.20527/kss.v3i2.4976

[ref35] Narimo S. Irawan E. P. (2018). Manajemen Ekstrakurikuler Hizbul Wathan Dalam Pengembangan Nilai-Nilai Karakter Di SMK. J. Manajemen Pendidikan 13, 210–215. doi: 10.23917/jmp.v13i2.7489

[ref36] Newman L. Dale A. (2005). The role of agency in sustainable local community development. Local Environ. 10, 477–486. doi: 10.1080/13549830500203121

[ref37] Nunes F. Mota C. P. Ferreira T. Schoon I. Matos P. M. (2023). Stability and change in adolescents’ sense of agency: contributions of sex, multiple risk, pandemic stress, and attachment to parents. J. Youth Adolesc. 52, 1374–1389. doi: 10.1007/s10964-023-01766-x, PMID: 36964433 PMC10038371

[ref38] Oberle E. Ji X. R. Guhn M. Schonert-Reichl K. A. Gadermann A. M. (2019). Benefits of extracurricular participation in early adolescence: associations with peer belonging and mental health. J. Youth Adolesc. 48, 2255–2270. doi: 10.1007/s10964-019-01110-2, PMID: 31440881

[ref39] Obradović J. Sulik M. J. Shaffer A. (2021). Learning to let go: parental over-engagement predicts poorer self-regulation in kindergartners. J. Fam. Psychol. 35, 1160–1170. doi: 10.1037/fam0000838, PMID: 33705178

[ref40] Oswell D. (2013). The agency of children: from family to global human rights. Cambridge: Cambridge University Press.

[ref41] Patle T. (2024). School extracurricural activity. Gurukul Int. Multidiscip. Res. J. XII, 377–384. doi: 10.69758/gimrj2406i8v12p046

[ref42] Rogers S. (2022). Play in the time of pandemic: children’s agency and lost learning. Education 50, 494–505. doi: 10.1080/03004279.2022.2052235, PMID: 40101104

[ref43] Schwarzer R. (2001). Stress, resources, and proactive coping. Appl. Psychol. 50, 370–408. doi: 10.1111/1464-0597.00063

[ref44] Sorokin P. S. (2023). The problem of “agency” through the prism of a new reality: conditions and perspectives. Sotsiologicheskie Issledovaniya 3, 103–114. doi: 10.31857/S013216250022927-2

[ref45] Sorokin P. S. Popova T. A. (2021). Classical and contemporary approaches to the study of solidarity: challenges and perspectives under Destructuration. RUDN J. Sociol. 21, 457–468. doi: 10.22363/2313-2272-2021-21-3-457-468

[ref46] Sorokin P. S. Popova T. A. (2022). Human capital qualities in responding to challenges for social policy in the context of De-structuration. J. Soc. Policy Stud. 20, 157–168. doi: 10.17323/727-0634-2022-20-1-157-168

[ref47] Syaharuddin S. Rahman A. M. Fitriyani R. (2020). Utilization of social community as learning resources on social studies. Kalimantan Soc. Stud. J. 1, 18–24. doi: 10.20527/kss.v1i1.1253

[ref48] Torrance S. Froese T. (2011). An inter-enactive approach to agency: participatory sense-making, dynamics, and sociality. Humana. Mente 15, 21–53.

[ref49] Trust T. Whalen J. (2020). Should teachers be trained in emergency remote teaching? Lessons learned from the COVID-19 pandemic. J. Technol. Teach. Educ. 28, 189–199. doi: 10.70725/307718pkpjuu

[ref50] Tysinger P. D. Tysinger J. A. Diamanduros T. D. (2016). Crisis events in K-12 online learning: educator perceptions and preparedness. Natl. Youth Risk J. 2, 41–48. doi: 10.20429/nyarj.2016.020104

[ref51] Udehn L. (2002). The changing face of methodological individualism. Annu. Rev. Sociol. 28, 479–507. doi: 10.1146/annurev.soc.28.110601.140938

[ref52] UNESCO. (2020). Education: from disruption to recovery. Available online at: https://www.unesco.org/en/covid-19/education-disruption-recovery (accessed June 13, 2024).

[ref53] Victor B. Fischer E. F. Cooil B. Vergara A. Mukolo A. Blevins M. (2013). Frustrated freedom: the effects of agency and wealth on well-being in rural Mozambique. World Dev. 47, 30–41. doi: 10.1016/j.worlddev.2013.02.005, PMID: 25125791 PMC4128575

[ref54] Weaver J. L. Swank J. M. (2021). Parents’ lived experiences with the COVID-19 pandemic. Fam. J. 29, 136–142. doi: 10.1177/1066480720969194

[ref55] Zimmerman B. J. Cleary T. J. (2006). “Adolescents’ development personal agency” in Adolescence and education: Self-efficacy beliefs of adolescents. eds. Pajares F. Urdan T. (Greenwich, CT: Information Age Publishing), 45–69.

